# Effects of Water-to-Cement and Sand-to-Binder Ratio on Mechanical and Drying Shrinkage Properties of Low-Carbon Mortar Containing Biochar Aggregate

**DOI:** 10.3390/ma18122750

**Published:** 2025-06-11

**Authors:** Shasha Chen, Junhui Zhang, Hao Yang

**Affiliations:** 1 Key Laboratory of Highway Engineering, Ministry of Education, Changsha University of Science & Technology, Changsha 410114, China; chenshasha@stu.csust.edu.cn (S.C.); hyang@csust.edu.cn (H.Y.); 2 School of Transportation, Changsha University of Science & Technology, Changsha 410114, China

**Keywords:** biochar aggregate, carbon neutrality, low-carbon mortar, sustainable waste management, eco-friendly materials, sustainable development

## Abstract

Biochar, serving as a carbon sequestration material, has garnered significant attention. In this study, the effects of water-to-cement (W/C) and sand-to-binder (S/B) ratio on the macroscopic mechanical properties, dry-shrinkage behavior, and water transport properties of biochar mortar, as well as the microstructure of the mortar, are described. The results indicate that the compressive strength of the mortar decreases gradually with increases in the S/B ratio, while its flexural strength increases gradually with increases in the S/B ratio. Meanwhile, with increases in W/C and S/B, the drying shrinkage rate decreases, and the extent of water loss tends to be comparable to the drying shrinkage rate. The water absorption of biochar mortar increases as the W/C and S/B ratios increase. This is also reflected in the depth of water ingress in biochar mortars, which increases significantly with rising W/C and S/B ratios. Moreover, the water absorption coefficients of different mortars vary significantly only in the first few hours, and their final water absorption coefficients and ingress depths are similar. The SEM results indicate that biochar can provide nucleation points for hydration products to form a unique binding mechanism between them and the cement matrix. In addition, when the sand-to-cement ratio reaches 1.15, biochar reduces CO_2_ emissions by 104.57 kg, and biochar mortar shows good potential for CO_2_ sequestration.

## 1. Introduction

At present, the construction industry ranks among the primary sources of CO_2_ emissions. This is because the production processes of cement and concrete entail substantial energy consumption. It is estimated that the production of cement emits a large amount of CO_2_ into the atmosphere. The CO_2_ produced by the production and use of cement accounts for approximately 7% of global greenhouse gas emissions [1]. In the future, the negative environmental impact of this will be further aggravated as the demand for construction materials surges alongside the rapid pace of global urbanization. One effective approach to curbing the carbon emissions linked to cementitious materials is to sequester stabilized carbon within the material itself while ensuring that the properties of the host matrix remain unaffected [2]. For sustainable cementitious composites, Biochar incorporation is a promising solution to promote carbon neutrality, as 1 ton of biochar can trap 2.0–2.6 tons of CO_2_ [3,4]. Biochar can be produced through the pyrolysis of various waste biomasses under limited oxygen conditions, subsequently stabilizing and storing carbon [5,6]. The renewable, widespread, and fast-growing nature of bio-based materials makes them a green alternative to natural gravel and sand in building materials [7]. Therefore, biochar can exert various beneficial effects, and its mechanism of action in cement-based materials warrants further investigation.

The use of biochar in cement composites has been mostly practiced by replacing cement with trace amounts of carbonized particles or by supplementing the cementitious material [8,9]. Gupta et al. [10] discussed the key points that make biochar a potential carbon sequestration additive for cement mortars, including preparation technology and the basic performances of biochar. Ahmad et al. [11] incorporated bamboo biochar into cement paste at dosages ranging from 0.05% to 0.20% by weight of cement. Compared with the reference sample, their results showed that the addition of 0.08% carbonized bamboo particles with a cement content of 0.08% increased the toughness and strength by 103% and 66%, respectively. The enhancement in toughness was ascribed to the crack-deformation mechanism induced by the biochar microparticles within the cement paste. Mishra et al. [4] suggest that the optimal amount of biochar (1%) slightly improves hydration and mechanical properties, but combination with mineral additives significantly improves the properties. The results show that biochar significantly improved the mechanical properties of concrete products. The use of biochar can sequester more carbon than alternative cementitious materials, and it has been shown that the use of biochar aggregate facilitates carbon sequestration by absorbing atmospheric carbon dioxide [12,13]. However, biochar is characterized by a loose and porous structure with high water absorption. Moreover, lowering the cement admixture significantly reduces the matrix strength. Therefore, it is particularly crucial to study the influence of the water–cement (W/C) ratio and sand–binder ratio (S/B) on the performance of biochar mortar.

Research has indicated that when the W/C of mortar is low (less than 0.20), this results in significant autogenous shrinkage. This is because the binder undergoes hydration while the capillaries remain unsaturated. Additionally, a low S/B impedes the shrinkage-inhibiting effect of aggregates and raises the volume percentage of the slurry, thereby eliminating the source that would otherwise mitigate shrinkage [14]. Shrinkage may cause the cracking of a specimen when subjected to internal or external constraints. Although biochar mortar can replenish a portion of the water lost during drying through the release of pre-absorbed water by shaped biochar, the volume stability of biochar mortar under different W/C and S/B ratios still warrants further investigation [15,16]. This is essential for determining appropriate W/C and S/B ratios to attain the desired mechanical properties, shrinkage resistance, and applicability.

The aim of this study is to reveal and elucidate the mechanism of the effect of different W/C and S/B ratios on biochar mortar, and to discuss their effects from the perspectives of mechanical properties and microstructure. The mechanical strength, drying shrinkage, and water loss of biochar mortar were experimentally determined, exploring the effects of biochar as fine aggregate on the mechanical properties of biochar mortar. Additionally, microscopic tests were conducted by SEM to investigate the microstructure between the interface of biochar and mixtures, and which will provide a valuable reference for the preparation of biochar-based materials.

## 2. Materials and Methods

### 2.1. Materials

**Figure 1:**
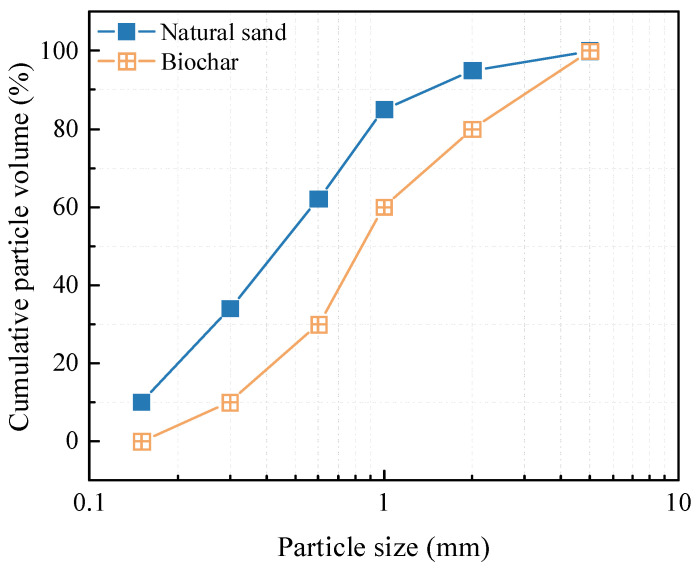
Particle size distribution of aggregates.

**Figure 2:**
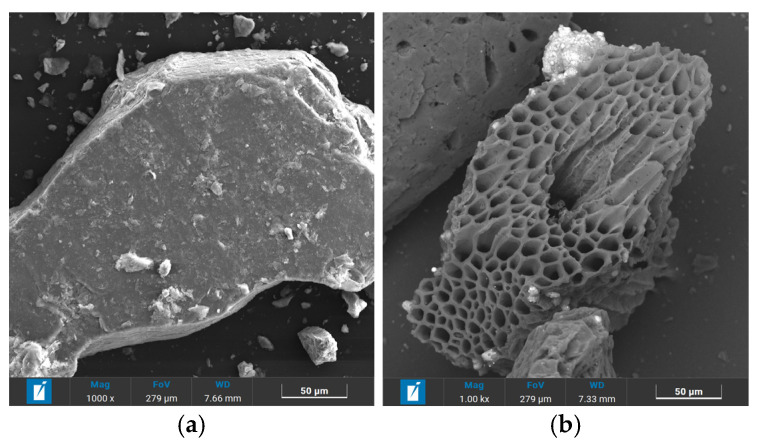
Photographs of samples. (**a**) Natural river sand. (**b**) Biochar.

The primary cementitious material was ordinary Portland cement (OPC) of class 42.5R from China Southern Cement Co., LTD, Shanghai, China which met the GB 175-2007 standard [17]. Locally sourced natural river sand (NS), categorized as Zone II was utilized as the fine aggregate. The biochar used in this study was produced from the pyrolysis of waste bamboo (BC) for 2 h at 500~700 °C using a warming speed of 10 °C m^−1^ under a limited supply of air [18]. The BC and NS were evaluated in accordance with the basic performance test methods of the (GB/T14684-2022) standard [19]. The particle size distribution of the biochar was 0–5 mm, as shown in Figure 1. Table 1 shows the properties of the NS and BC. Compared with the NS, the apparent density of the BC was lower than that of the NS, but its water absorption was much greater than that of the NS. Figure 2 displays the appearance of the natural river sand and biochar. From the SEM image, it can be observed that the BC possessed an irregular sawtooth shape and showed a narrow lotus root shape. The NS had a regular shape with a smooth surface devoid of obvious pores. Table 2 provides an overview of the findings.

### 2.2. Mixture Design and Sample Preparation

#### 2.2.1. Mixture Design

Eight mixtures of mortars were designed in this study, including different water–cement ratios (W/C) and sand–binder ratios (S/B) (Table 2). The volume dosage of biochar was kept constant at 20% of the total volume of fine aggregate. In addition, four sets of samples were prepared in terms of S/B ratio. Based on the reference group (i.e., WC0.3), additional sample groups were prepared by adjusting the S/B ratios to 0.46, 0.69, and 1.15.

The fragility and high water absorption of biochar limit its use in cement-based materials at high dosages. To address the adverse impact of biochar on the workability of mortar, the water in the mixture was measured as the sum of the water-mixed (water-m) and water-added (water-a) components. [20]. Since the water absorption rate of biochar could not reach saturation during mixing, the method of calculating saturated water absorption may lead to changes in the W/C ratio (shown as Equation (1)). Therefore, a method for calculating additional moisture was chosen to achieve a 30% moisture condition for the biochar (shown as Equation (2)).

(1) $$M_{aw}{\,}_{-100\%}=\left(m_{w}-C_{w}\right)\times m_{fa}$$

(2) $$M_{aw}{\,}_{-30\%}=(30\%\times m_{w}-C_{w})\times m_{fa}$$

where *M_aw-100%_* represents the weight of water added to achieve a 100% moisture content. *M_aw-30%_* represents the weight of additional water aiming to 30% moisture content. *m_w_* is the water absorption ratio of aggregates. *C_w_* is the water absorption ratio of aggregates.

#### 2.2.2. Specimens Preparation

During mixing, all aggregates, including the oven-dried natural river sand and biochar, were added to a mortar mixer together with 1/3 of the total water and mixed for 1 min. Then, all the cement was added and mixed for 1 min. Finally, the superplasticizer, together with the rest of the water (2/3 of the total), was slowly added to the mixture and then mixed for 2 min [21,22]. For each mixture, 18 cubes of 40 × 40 × 40 mm^3^ and 40 × 40 × 160 mm^3^ were cast. The mixing and casting of the mortar were conducted under ambient conditions (25 ± 2 °C, ≥60% RH). The specimens adopted two curing mechanisms. The specimens for air curing were demolded after 24 h and then stored in laboratory room (25 ± 10 °C, 50 ± 20% RH) for 27 d. The specimens for moist curing were cured in a container filled with a saturated calcium hydroxide solution to prevent the leaching of calcium hydroxide from affecting the hydration process of the cement [20]. In this work, the corresponding size of the testing samples for the various experiments are shown in Table 3. Table 3 shows the number of samples corresponding to each mixing ratio in each experiment, illustrating that the results data in the following tables and figures are the average test results of multiple samples.

### 2.3. Testing Methods

#### 2.3.1. Mechanical Properties

The mechanical properties were measured by loading specimens at a rate of 3000 kN in a hydraulic pressure testing machine. The compressive strength of the mortar at 28 days was determined under a loading rate of 2.4 kN/s, while the flexural strength was measured at a loading rate of 0.05 kN/s, in accordance with Chinese standard (JTG 3420-2020) [23].

#### 2.3.2. Drying Shrinkage and Mass Loss

The drying shrinkage test was conducted using prismatic mortar, and the lengths of the mortar samples before and after exposure under dry conditions were determined by a comparator. The length of mortar samples before and after drying was employed to measure the lengths of specimens, including the initial length and changed lengths exposed to dry conditions for 3 days, 5 days, 7 days, 10 days, 14 days, 18 days, 21 days, 24 days, and 28 days. The relative temperature and humidity in the drying shrinkage test were 20 ± 3 °C and 50 ± 5, respectively. The drying shrinkage and resulting mass loss were tested in parallel with the drying shrinkage test.

#### 2.3.3. Water Transport Properties and Total Porosity

The durability of cement-based materials is influenced by their water absorption and total porosity, which are commonly used indicators for assessing the difficulty of water infiltration [24,25]. To ensure the complete drying of the mortar, the cubic block was cured for 28 days and then placed in a 60 °C drying oven for 72 h to achieve thorough drying. We left the bottom of the mortar sample exposed as a water infiltration channel and sealed the other surfaces to ensure that water could enter the sand from one direction only. A bracket was then put into a flat container, and the treated mortars were then tested on the bracket, with their testing face down, pouring water into the container slowly until the water level was 5 ± 2 mm above the bottom of the sample. A holder was then placed into the container and the surface-treated mortar was placed on the holder for testing. With the test surface facing down, water was slowly poured into the container until the water level was 5 ± 2 mm above the bottom of the sample. At designated time intervals (i.e., 0.003, 0.02, 0.5, 1, 2, 4, 8, 12, 24, 48, and 72 h), the samples were weighed after drying their surfaces with a wet towel. The humidity of the test environment was 60 ± 5%. Equation (3) provides the formula for the determination of water absorption. After neglecting the effect of gravity on the water absorption of mortar, Equations (4) and (5) can further characterize the time-varying characteristics of water absorption, where Equation (5) gives the slope of the regression line of the linear correlation as the time-varying capillary absorption coefficient *C_w_* (g/(m^2^h^1/2^)), which is calculated by fitting the time-varying curves of water absorption at the first 2 h, reflecting the transport capacity of water in cement-based materials [26]. Furthermore, the total porosity of the mortars with different biochar species, biochar particle sizes, and biochar dosages were determined by the vacuum saturation method. The total porosity was calculated using Equation (6).

(3) $$Q=\left(M_{1}-M_{0}\right)/A$$

(4) $$Q=C_{w}\sqrt{t}$$

(5) $$C_{w}(t)=\frac{dQ}{d\sqrt{t}}=C_{i}e^{-a\sqrt{t}}$$

(6) $$P=\frac{\left(Q_{sat}-Q_{dry}\right)}{\left(Q_{sat}-Q_{wat}\right)}\times 100$$

where *Q* represents the water absorption amount (g/m^2^), *M*_0_ and *M*_1_ are the sample weight before and after water absorption (g), *A* represents the absorption area (40 mm × 40 mm), *C_w_* is the capillary absorption coefficient (g/(m^2^h^1/2^)), *t* is the water absorption time (h), and *Q_sat_*, *Q_dry_*, and *Q_wat_* are the saturated mass in air, dry mass, and saturated mass in water of the samples, respectively.

#### 2.3.4. Microscopic Performance

Scanning electron microscope (SEM) analysis:

Scanning electron microscopy (SEM) was performed on hydrated mortar at the age of 28 days using a Zeiss (Gemini 500) electron microscope (Zeiss, Oberkochen, Germany). For backscattered electron (BSE) image analysis, slices impregnated in epoxy resin were cut out at the middle of the cubic specimen blocks and dried in a vacuum dryer for 48 h. The polished samples were sprayed with gold and subjected to SEM at an accelerating voltage of 20 kV.

## 3. Results and Discussion

### 3.1. Mechanical Strengths

#### 3.1.1. Effects of Different Conditions on Mechanical Strength

**Figure 3:**
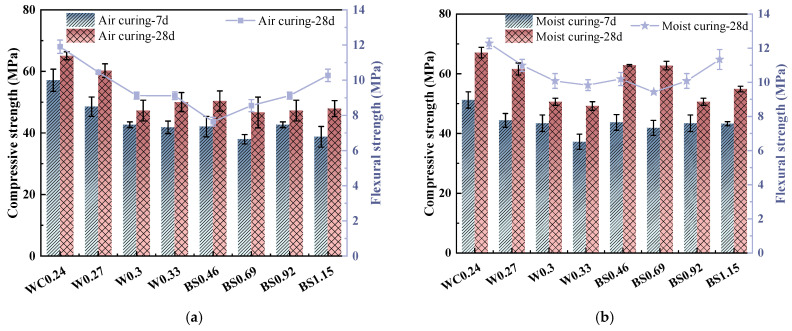
Compressive strength of biochar mortars with different curing conditions. (**a**) Air curing. (**b**) Moist curing.

Figure 3 displays the mechanical strengths of the biochar mortars with different curing conditions. As shown in Figure 3, the mechanical strength of the biochar mortar under air-curing conditions was lower than that under wet-curing conditions, indicating that wet-curing conditions favored the strength development of biochar mortar. Similarly, as the curing age increased, the compressive strength increased, which was a result of cement hydration and was not affected by the curing environment [27]. The results showed that the mechanical properties of the biochar mortar were better in moist-curing conditions compared to air-curing conditions. This is due to the fact that the porous biochar increases the water absorption of the biochar mortar, making it effective in replenishing the drying water loss of the mortar under natural environmental conditions [28].

The mechanical strength decreases gradually with the increasing W/C ratio, which is consistent with the previous literature [29]. Obviously, the mechanical strength of the material experiences a substantial decline at relatively high W/C ratios. Nevertheless, when the W/C ratio exceeds 0.3, the rate of strength reduction gradually diminishes. Regardless of the maintenance conditions, the compressive strength of the mortar with an increase of S/B ratio showed a tendency to first decrease and then increase. However, the flexural strength of the mortar increases gradually with the increase in the S/B ratio, which is consistent with the findings of Ye et al. [14]. The inclusion of sand in mixtures means including strong particle distribution in the system, which helps to improve the mechanical properties of mortars [30]. Due to the large effect of maintenance conditions on the mechanical strength of biochar mortar, it is recommended that porous biochar mortar materials are not air-curing [18].

#### 3.1.2. Compressive and Flexural Strengths

**Figure 4:**
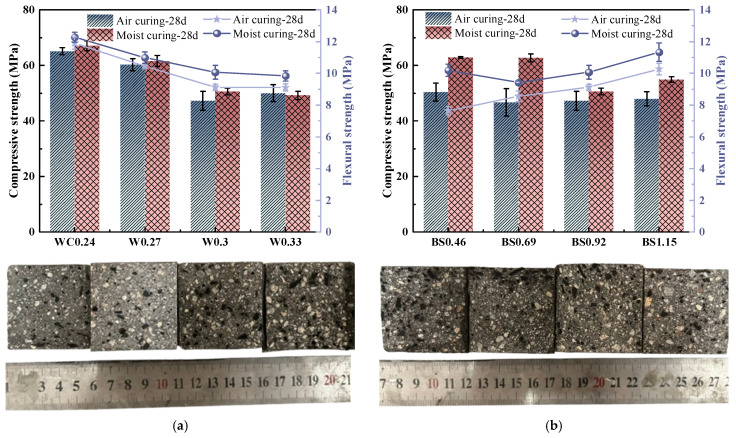
Mechanical strength of biochar mortar. (**a**) Different W/C. (**b**) Different S/B.

The average results for the compressive and flexural strength (*f*_cu_ and *f*_f_, respectively) of three samples, along with each value’s corresponding standard deviation (S.D.), are reported in Table 4. Figure 4 shows the mechanical performance of the biochar mortar, and the difference in mechanical strength was more obvious when high W/C and S/B ratios were used. As shown in Figure 4a, mechanical strength decreases with an increasing W/C ratio. By comparison with WC0.24, the WC0.27, WC0.3, and WC0.33 samples show 8.2%, 24.6%, and 26.7% decreases in compressive strength, and are 10.6%, 17.9%, and 20.3% lower in flexural strength, respectively. It is not difficult to find that the mechanical strength of biochar mortar deteriorated significantly at high water–cement ratios. This is due to the fact that the increase in water consumption significantly reduces the mass fraction of cement, which leads to a decrease in the degree of hydration [31].

Figure 4b further demonstrates the effect of different S/B ratios on the mechanical properties of biochar mortar. The results illustrate that the compressive strength of the mortar decreases gradually with the increase in the S/B ratio, while its flexural strength increases gradually with the increase in the S/B ratio. The dosage of biochar gradually increased with the increase in the S/B ratio, leading to a decrease in the mechanical properties of the mortar, which was attributed to the increase in the total porosity, although biochar incorporation improved cement hydration. Moreover, a large amount of biochar is not conducive to the development of interfacial transition zone (ITZ) strength in the mortar [5,32]. Although biochar is more sustainable for the development of cementitious materials, the excessive addition of biochar aggregate leads to a reduction in mechanical strength. However, the increase in flexural strength may be due to the physical action of the biochar; i.e., the angular and hollow surface of the biochar enhances its adhesion to the mortar matrix [33]. In addition, biochar may provide more nucleation sites and thus promote the generation of hydration products [34,35].

### 3.2. Drying Shrinkage and Water Loss Behavior

**Figure 5:**
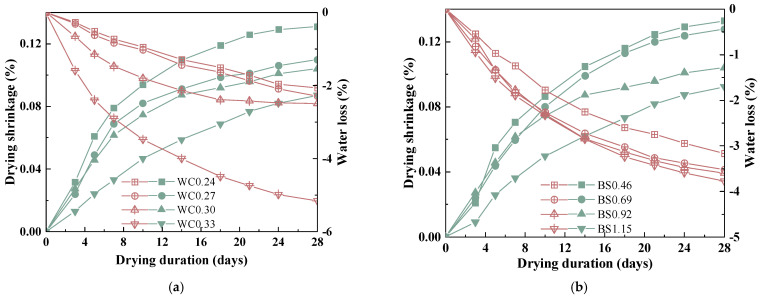
Drying shrinkage and water loss of biochar mortars. (**a**) W/C. (**b**) S/B.

The drying and shrinking of mortar is closely related to humidity and interconnected micropores in its matrix, especially in biochar mortar with a porous structure [6,36]. Also, previous studies have shown a positive correlation between mass loss and drying shrinkage [37]. The drying shrinkage and percentage of water loss in biochar mortars is shown in Figure 5. The drying shrinkage in biochar mortar exhibits an initial significant increase, followed by a gradual slowing down over time. The mixing in of biochar decreased the drying shrinkage, and the 28-day drying shrinkages of WC0.27, WC0.3, and WC0.33 were 15.4%, 23.1%, and 30.8% lower than that of WC0.24. When the w/c ratio was greater than 0.3, the mortars had sufficient mixing water inside so as to prevent the drying shrinkage effect. Interestingly, the maximum drying shrinkage of the biochar mortar was significantly lower than that of the recycled mortar [38], UHPC [39], recycled concrete [40], and natural mortar [41]. This is related to the fact that porous biochar has an internal curing effect. According to Lin et al. [37], the addition of biochar reduces the drying shrinkage of the mortars because biochar reduces the loss of water in the mortar matrix. Figure 5b depicts that an increase in S/B can lower the tendency of drying shrinkage. An increase in S/B can lower the volume percentage of cement paste and facilitate the skeleton effect of aggregate, thus decreasing the magnitude of drying shrinkage [14]. In addition, the percentage of mass loss exhibited a similar trend with drying shrinkage value. Due to the additional water required to achieve the saturated surface drying of the BC before the preparation of biochar mortar, the ever-increasing S/B ratio causes a rise in free water content. Thus, compared to natural mortar, biochar mortar has a mitigating effect on drying shrinkage due to early water absorption in the pores of the biochar and the subsequent desorption during drying shrinkage [42].

**Figure 6:**
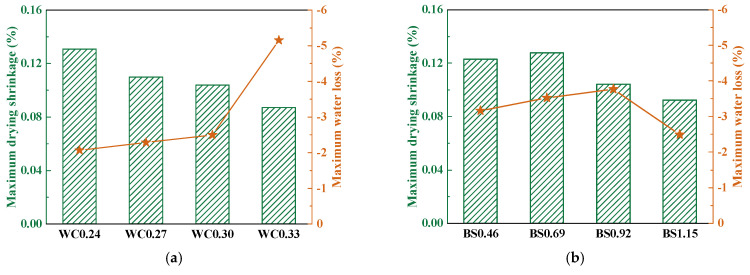
Maximum drying shrinkage and water loss. (**a**) W/C. (**b**) S/B.

As shown in Figure 6a, with the increase in W/C, the drying shrinkage decreased accordingly. However, water loss decreased with increasing W/C ratios. A reduced W/C ratio improved the cement volume proportion per unit mortar volume, thereby enhancing capillary pore undersaturation. This phenomenon aggravated the drying shrinkage of the samples and subsequently induced significant water loss [43]. Under high-W/C-ratio conditions, the slope of the water loss curve was significantly amplified, reflecting intensified capillary-driven water migration. The maximum drying shrinkage of the mortar corresponds to the result of the maximum water loss. Larger drying shrinkage corresponds to smaller water loss, and conversely smaller drying shrinkage corresponds to larger water loss. This is similar to previous results [21]. Furthermore, the drying shrinkage first increased and then decreased with increasing S/B ratios (Figure 6b). This is due to the increase in S/B reducing chemical shrinkage and autogenous shrinkage [44]. The enhancement of S/B can decrease the cement paste percentage per unit volume of mortar, which is the source of chemical shrinkage and autogenous shrinkage, so an increase in S/B reduces the magnitude of chemical shrinkage and autogenous shrinkage [45].

### 3.3. Water Transport Properties and Porosity

**Figure 7:**
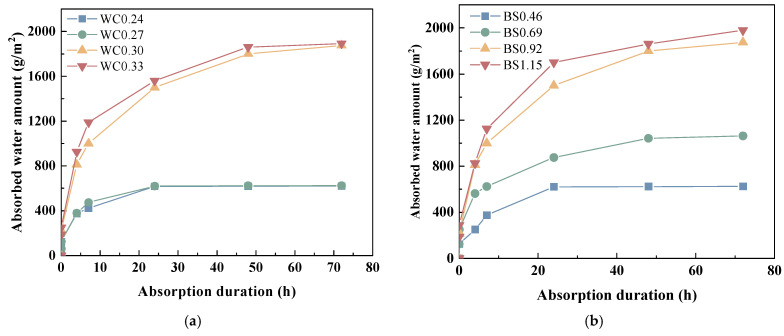
The water absorption curves of biochar mortar. (**a**) W/C. (**b**) S/B.

Figure 7a exhibits the water absorption curve of the biochar mortars. As can be seen, increasing the W/C ratio increased the water absorption. This is because a decrease in the W/C ratio affects the workability of the mortar by introducing more excess initial porosity. The water absorptions of WC0.27, WC0.3, and WC0.33 were 11.8%, 202.4%, and 205.2% higher than that of WC0.24. At low W/C ratios, the increase in the water absorption of biochar mortar was not significant, but when the W/C ratio was greater than 0.3, its water absorption exhibited a substantial increase. This is because biochar contains a large number of pores, and the high water to ash ratio provides a greater source of moisture for the biochar [46]. As shown in Figure 7b, the water absorption of the biochar mortar increases with the S/B ratio. This can be attributed to the large number of macro-tubular pores in the biochar mortar, which provide access to water intake, while the high water absorption of the biochar further enhances the water intake efficiency of the mortar [47].

**Figure 8:**
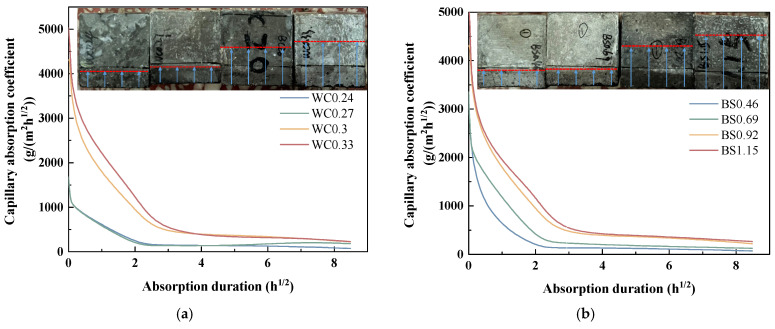
Variation in capillary absorption coefficient of biochar mortar over time. (**a**) W/C. (**b**) S/B.

Figure 8a presents the variation in capillary absorption coefficient of the biochar mortars over time. It is shown that both the initial *Cw* and time-varying *Cw* generally increased with increasing W/C and S/B ratios. The immersion depth of biochar mortar increases significantly with increases in the W/C and S/B ratios. Moreover, the *Cw* of the different mortars only differed significantly in the first few hours, and their final coefficients and water ingress depth were similar.

**Figure 9:**
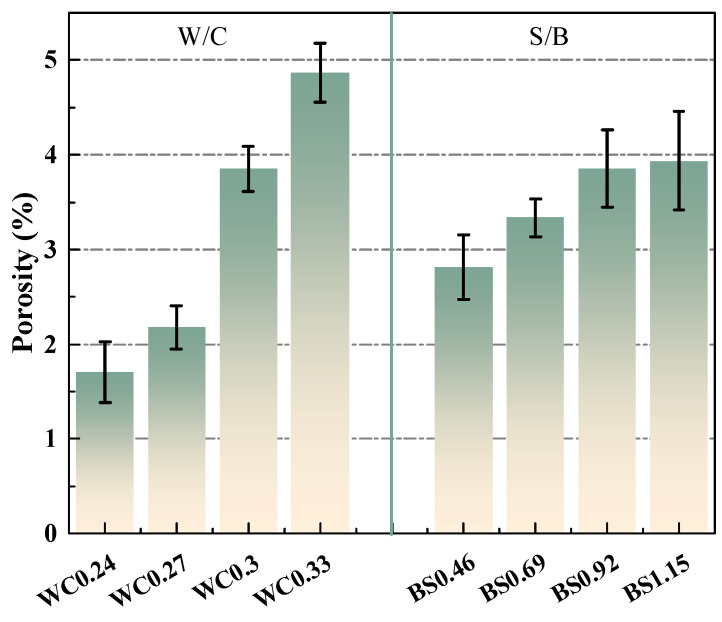
Total porosity of biochar mortars.

**Figure 10:**
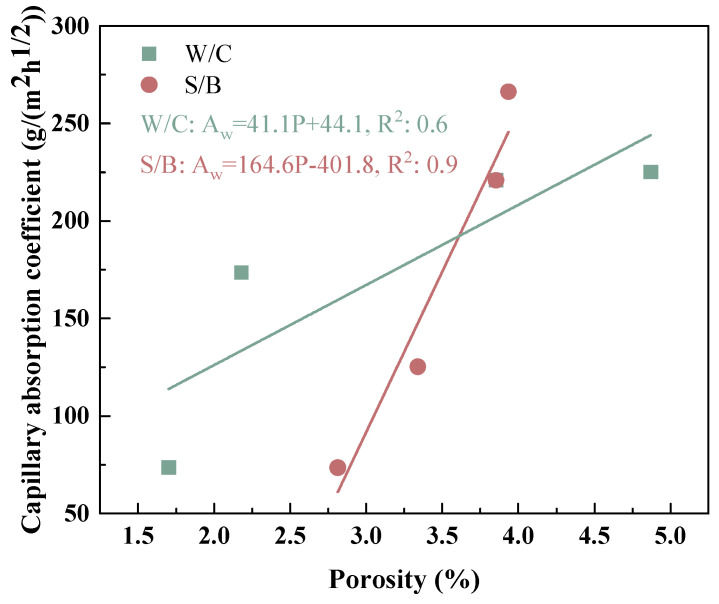
Relationship between water transport properties and porosity of biochar mortars.

Figure 9 shows the total porosity of the biochar mortars. As a whole, the porosity increased with the increase in W/C and S/B ratio, which is consistent with the results of mechanical strength in Section 3.1. Especially when the water–cement ratio exceeds 0.3, the porosity of the biochar mortar increases sharply. For different S/B ratios, the overall porosity of the mortar increased due to the introduction of more pores through the increase in biochar dosage. Nonetheless, with the increment of the S/W ratio, the progressive increase in the porosity of the mortar becomes less conspicuous. This means that the addition of an appropriate amount of biochar can form a continuous gradation with natural river sand, making the mortar denser and more favorable to the development of mortar strength [48]. It is worth noting that the effect of the water–cement ratio on porosity is more significant than that of the sand–cement ratio, which provides a favorable reference for the design of the proportion of biochar mortar. By correlating the water transport behavior of the biochar mortar, Figure 10 further illustrates that the capillary absorption coefficient is proportional to the total porosity.

### 3.4. Microproperties of Cement Paste

#### Partial Bonding Mechanisms

**Figure 11:**
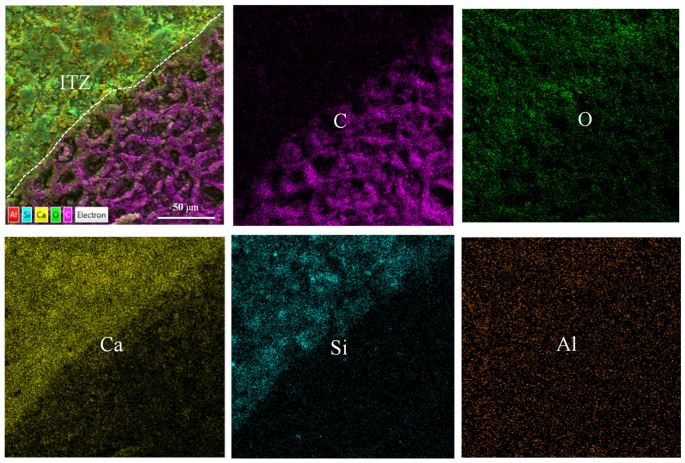
Partial bonding features of biochar and cement matrix.

**Figure 12:**
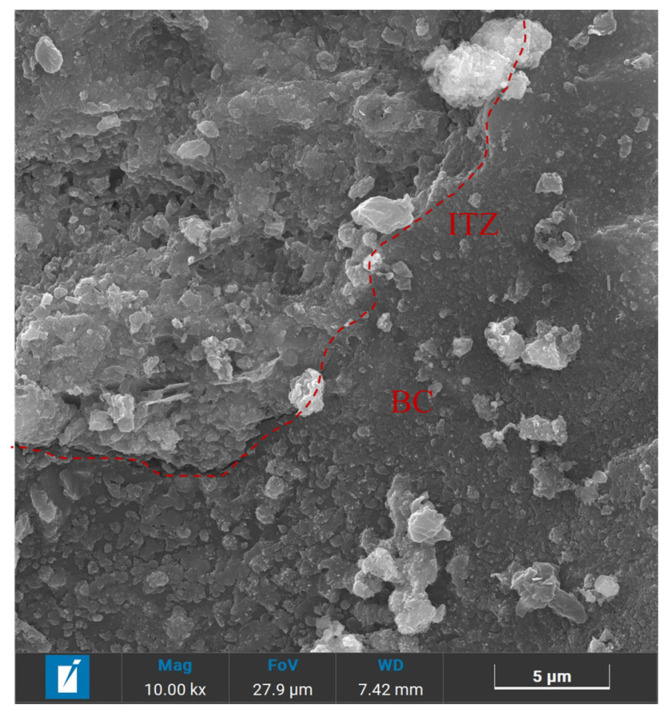
SEM image of further-enlarged ITZ.

Figure 11 reveals the partial bonding characteristics of the biochar to the matrix. The BSEM images provide valuable insights for observing the morphology and composition characteristics of relevant regions. Compositional information (i.e., average atomic number) of the regions of interest can be analyzed by EDX surface scanning, where brighter regions indicate heavier or denser components (i.e., higher average atomic numbers) [49]. A clear interfacial transition zone (ITZ) was observed in the specimens of biochar mortar. This is because the loose and porous surface of the biochar provided nucleation sites for the hydration reaction of the Portland cement, resulting in the formation of a denser ITZ structure [29,47]. Figure 12 shows that the brightest region corresponds to calcium hydroxide (CH), while the gray region between the biochar and CH represents a layer of C-S-H gel. Additionally, fewer microcracks at the ITZ can be seen, suggesting improved mechanical interlocking and adhesion. This is in agreement with the findings of Zhu et al. [50]. This is because the calcium ions in the cement can act as a bridge to the ITZ and provide a nucleation site for the growth of CH. Consequently, a distinctive bonding mechanism is established between the biochar and the cement matrix via calcium hydroxide (CH) or a stratum of calcium-silicate-hydrate (C-S-H) gel [50].

### 3.5. CO_2_ Footprint Performance of Biochar Mortar

Since the different W/C ratio samples only adjust the amount of water added and do not affect the overall carbon emissions, this section only discusses the different S/B ratio samples. Carbon emission analysis is generally based on process-based carbon emission analysis methods for statistics [32]. Due to its convenience and high reliability in LCA frameworks, the process-based approach has been widely used in the quantification and analysis of carbon emissions from materials production [51]. The CO_2_ emissions of the different mortars were calculated by the product of material consumption (*M*) and carbon emission coefficient (*C*_f_), as shown in Equation (5):

(7) $$C\mathrm{arbon}\;\mathrm{emission}=\sum_{i=1}^{n}M_{i}C_{fi}$$

where carbon emission is the total carbon emission of the production, M_i_ represents the material consumption, and C_fi_ represents the emission coefficient of carbon emission for material consumption.

**Figure 13:**
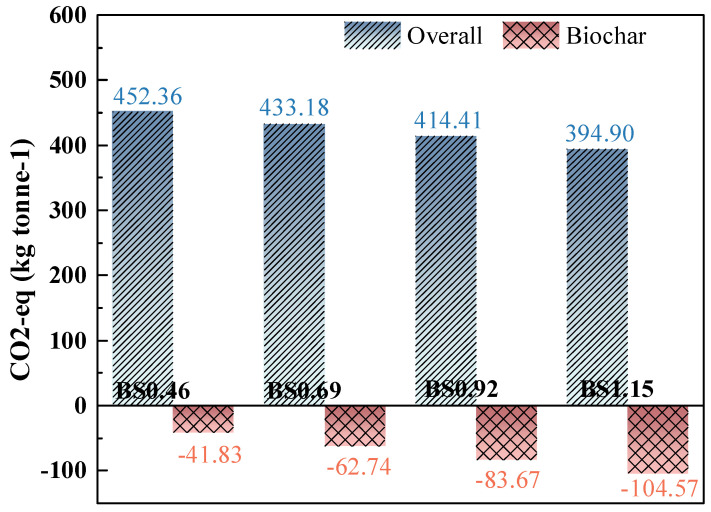
CO_2_ emissions of biochar mortar with different W/C and S/B ratios.

The CO_2_ emissions of the biochar mortars are demonstrated in Figure 13. The results demonstrated that the incorporation of biochar significantly reduced CO_2_ emissions, which is expected to contribute to the production of low-carbon mortar. As we can see from Table 5, cement releases large amounts of carbon dioxide during production and use, which is not conducive to sustainable development. The incorporation of biocarbon facilitates the development of low-carbon mortar. The CO_2_ emissions of the biochar mortars decreased significantly with the increase in biochar content. When the sand-to-cement ratio reaches 1.15, biochar reduces CO_2_ emissions by 104.57 kg. This results in an 87.3% reduction in overall mortar carbon emissions compared to BS1.15. Therefore, this result is in contrast to earlier studies, where the replacement of natural aggregates with biochar produced CO_2_ emissions that were between 6.2% [32] and 46.6% [52] lower than ordinary concrete/mortars. Therefore, biochar mortar shows good CO_2_ sequestration potential.

## 4. Conclusions

This study developed an innovative method to promote the use of different W/C and S/B ratios for the production of biochar mortar. The influence of biochar on the mechanical properties, drying shrinkage behavior, water transport properties, and micro-composition and -structure of mortar were determined. The environmental impact of mortar products with and without biochar were evaluated. The following conclusions can be obtained:

1. The mechanical strength decreases with an increasing W/C ratio. And the compressive strength of the mortar decreases gradually with increases in the S/B ratio, while its flexural strength increases gradually with increases in the S/B ratio. Although biochar is more sustainable for the development of cementitious materials, the excessive addition of biochar aggregate leads to reductions in mechanical strength.
2. The percentage of mass loss exhibits a similar trend alongside drying shrinkage value. Due to the additional water required to achieve the saturated surface drying of the BC before the preparation of biochar mortar, the ever-increasing S/B ratio causes a rise in free water content. Thus, compared to natural mortar, biochar mortar has a mitigating effect on drying shrinkage due to early water absorption in the pores of the biochar and the subsequent desorption during drying shrinkage.
3. The water absorption of the biochar mortar increases with the W/C and S/B ratios. We also display the water ingress depth in biochar mortar, and the water ingress depth in mortar obviously increased with increases in W/C and S/B ratio. Moreover, the *C*_w_ of the different mortars differed significantly only in the first few hours, and their final coefficients and water ingress depth were similar.
4. Biochar can provide nucleation sites for hydration products, allowing a unique binding mechanism between them and the cement matrix, i.e., through CH or a layer of C-S-H gel. When the S/B ratio reaches 1.15, the biochar can result in a reduction 104.57 kg of CO2eq, and biochar mortar shows good potential for CO2eq sequestration.

## Figures and Tables

**Table 1:** Characteristics of fine aggregates.

Type	Apparent Density (kg/m^3^)	Water Absorption (%)	Fineness Modulus	Moisture Content (%)
NS	2630	1.1	3.2	0.5
BC	1540	63	2.8	5.9

**Table 2:** Mix proportions of mortar.

Mix		Material Composition (kg/m^3^)						Slumps (mm)
		Cement	NS	Biochar	W-m	W-a	SP	
W/C	WC0.24	667.6	534.1	78.2	160.2	0	2.3	190
	WC0.27	667.6	534.1	78.2	180.3	14.8	2.3	210
	WC0.3	667.6	534.1	78.2	200.3	14.8	2.3	225
	WC0.33	667.6	534.1	78.2	220.3	14.8	2.3	235
S/B	BS0.46	667.6	267.0	39.1	200.3	7.4	2.3	225
	BS0.69	667.6	400.6	58.6	200.3	11.1	2.3	235
	BS0.92	667.6	534.1	78.2	200.3	14.8	2.3	220
	BS1.15	667.6	667.6	97.7	200.3	18.5	2.3	220

Notes: The label WC0.24 and BS0.46 indicate that the W/C and S/B of the mixture are 0.24 and 0.46, respectively. The label SP represents a polycarboxylate superplasticizer.

**Table 3:** Testing samples.

Information	Testing and Determinations				
	Compressive Strength (Air Curing)	Compressive Strength (Moist Curing)	Flexural Strength	Drying Shrinkage	Water Absorption
Size (mm)	40 × 40 × 40	40 × 40 × 40	40 × 40 × 160	40 × 40 × 160	40 × 40 × 40
Number	6 samples	6 samples	6 samples	3 samples	3 samples

**Table 4:** Mechanical properties of biochar mortars.

Mix	7d *f*cu (MPa)	S.D. (MPa)	28d *f*cu (MPa)	S.D. (MPa)	28d *f*_f_ (MPa)	S.D. (MPa)
WC0.24	57.13	1.28	65.08	3.63	11.91	0.38
W0.27	48.54	2.19	60.25	3.15	10.45	0.07
W0.3	42.67	3.39	47.27	0.92	9.125	0.20
W0.33	41.83	3.10	50.02	2.03	9.12	0.21
BS0.46	42.06	3.26	50.40	3.31	7.64	0.25
BS0.69	37.98	4.99	46.67	1.53	8.55	0.34
BS0.92	42.67	3.39	47.27	0.92	9.13	0.20
BS1.15	38.75	2.58	47.94	3.36	10.27	0.35

Note: *f*_cu_ and *f*_f_ represent the compressive and flexural strengths of the mortar, respectively.

**Table 5:** COE factors of materials.

Materials	Emission Factor	Unit	Reference
OPC	735	kg CO_2_ eq/t	[53]
Natural river sand	13	kg CO_2_ eq/t	[54]
Water	0.168	kg CO_2_ eq/t	[53]
SP	188	kg CO_2_ eq/t	[4]
Biochar	−1070	kg CO_2_ eq/t	[55]

## Data Availability

The original contributions presented in this study are included in the article. Further inquiries can be directed to the corresponding author.
